# IGFBP-1 expression is reduced in human type 2 diabetic glomeruli and modulates β1-integrin/FAK signalling in human podocytes

**DOI:** 10.1007/s00125-021-05427-1

**Published:** 2021-03-24

**Authors:** Abigail C. Lay, Lorna J. Hale, Holly Stowell-Connolly, Robert J. P. Pope, Viji Nair, Wenjun Ju, Eva Marquez, Ruth Rollason, Jenny A. Hurcombe, Bryony Hayes, Timothy Roberts, Lawrence Gillam, Jonathan Allington, Robert G. Nelson, Matthias Kretzler, Jeff M. P. Holly, Claire M. Perks, Craig A. McArdle, Gavin I. Welsh, Richard J. M. Coward

**Affiliations:** 1 Bristol Renal, Bristol Medical School, University of Bristol, Bristol, UK; 2 Translational Health Sciences, Bristol Medical School, University of Bristol, Bristol, UK; 3 Division of Nephrology, Department of Internal Medicine, University of Michigan, Ann Arbor, MI USA; 4 Department of Computational Medicine and Bioinformatics, University of Michigan, Ann Arbor, MI USA; 5 National Institute of Diabetes and Digestive and Kidney Diseases, National Institutes of Health, Phoenix, AZ USA; 6 IGFs and Metabolic Endocrinology Group, Bristol Medical School, University of Bristol, Bristol, UK

**Keywords:** Adhesion, Diabetic nephropathy, FAK, Fkhr, FoxO1, Glomerulus, IGFBP-1, β1-integrin, Motility, Podocyte

## Abstract

**Aims/hypothesis:**

Podocyte loss or injury is one of the earliest features observed in the pathogenesis of diabetic kidney disease (DKD), which is the leading cause of end-stage renal failure worldwide. Dysfunction in the IGF axis, including in IGF binding proteins (IGFBPs), is associated with DKD, particularly in the early stages of disease progression. The aim of this study was to investigate the potential roles of IGFBPs in the development of type 2 DKD, focusing on podocytes.

**Methods:**

*IGFBP* expression was analysed in the Pima DKD cohort, alongside data from the Nephroseq database, and in ex vivo human glomeruli. Conditionally immortalised human podocytes and glomerular endothelial cells were studied in vitro, where IGFBP-1 expression was analysed using quantitative PCR and ELISAs. Cell responses to IGFBPs were investigated using migration, cell survival and adhesion assays; electrical cell-substrate impedance sensing; western blotting; and high-content automated imaging.

**Results:**

Data from the Pima DKD cohort and from the Nephroseq database demonstrated a significant reduction in glomerular *IGFBP-1* in the early stages of human type 2 DKD. In the glomerulus, IGFBP-1 was predominantly expressed in podocytes and controlled by phosphoinositide 3-kinase (PI3K)–forkhead box O1 (FoxO1) activity. In vitro*,* IGFBP-1 signalled to podocytes via β1-integrins, resulting in increased phosphorylation of focal-adhesion kinase (FAK), increasing podocyte motility, adhesion, electrical resistance across the adhesive cell layer and cell viability.

**Conclusions/interpretation:**

This work identifies a novel role for IGFBP-1 in the regulation of podocyte function and that the glomerular expression of *IGFBP-1* is reduced in the early stages of type 2 DKD, via reduced FoxO1 activity. Thus, we hypothesise that strategies to maintain glomerular IGFBP-1 levels may be beneficial in maintaining podocyte function early in DKD.

**Graphical abstract:**

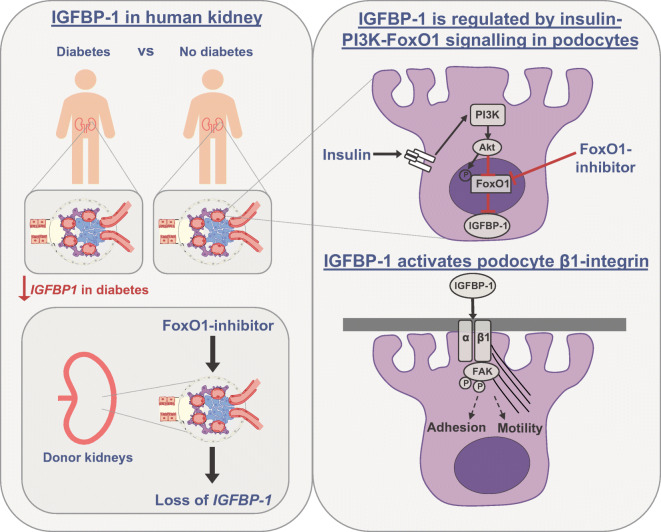

**Supplementary Information:**

The online version contains peer-reviewed but unedited supplementary material available at 10.1007/s00125-021-05427-1.

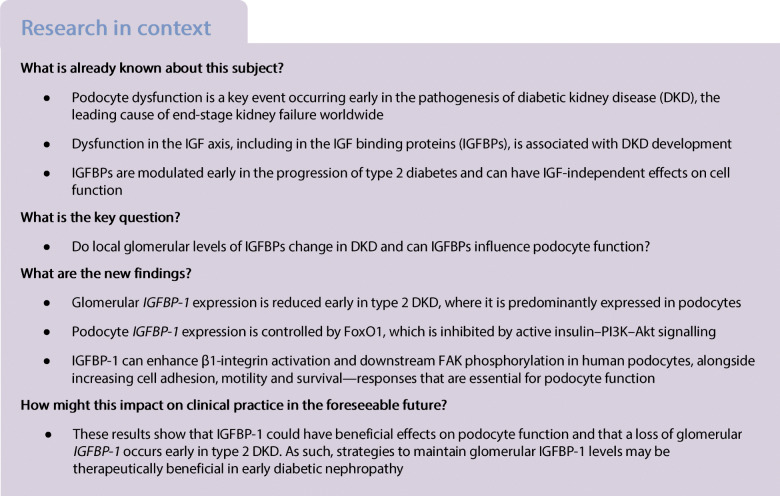



## Introduction

Diabetic kidney disease (DKD) occurs in approximately one-third of diabetic individuals and is the leading cause of end-stage renal failure worldwide. Albuminuria often presents early during the development of DKD and is an important risk factor for the progression to both end-stage renal failure and cardiovascular disease [1].

Podocytes are highly specialised, terminally differentiated epithelial cells which line the urinary side of the glomerular basement membrane (GBM) in the kidney. These cells have essential roles in filtration barrier maintenance and, as such, podocyte loss or injury is a major cause of albuminuria in numerous settings, including DKD. Both the effacement of podocyte foot processes and reduction in podocyte number or density (as a result of cell detachment and apoptosis) occur early in the pathogenesis of DKD, as well as predicting the progression of DKD [2, 3]. Thus, further understanding of the mechanisms involved in podocyte dysfunction in the setting of DKD is desirable.

The mammalian IGF axis comprises IGF-I and -II; their respective receptors, the IGF-I receptor (IGF-IR) and IGF-II/mannose-6-phosphate receptor (IGF-IIR/M6PR); and a family of six IGF binding proteins (IGFBP-1–6). Dysregulation of IGF signalling is associated with several metabolic conditions including the development and progression of DKD, particularly in the early stages of disease [4]. IGFBPs have also been linked to the pathogenesis of albuminuric renal disease [5–7].

Although the primary functions of the IGFBPs were once thought to be binding IGF-I and -II within the circulation, prolonging their half-life as well as regulating their passage into tissues, it is now well recognised that IGFBPs can bind to the cell surface, exerting cell-specific, IGF-independent effects on cell growth, differentiation and survival [8–10].

While it is known that both IGF-I and IGF-II signalling can directly influence podocyte biology [11, 12], the roles of IGFBPs in this context are not well understood. However, there is evidence that podocytes can also respond to IGFBP stimulation [5, 6]. The effects of IGFBPs in podocytes in the context of DKD have not been reported. In this study, we explored the local, glomerular production of IGFBPs in DKD and the IGF-independent effects of IGFBPs on podocytes.

## Methods

### Human samples

*IGFBP* expression was analysed in pre-collected gene expression data from the Pima DKD cohort, alongside existing expression data in the Nephroseq database. Background information from the Pima DKD study is as follows: Protocol human kidney biopsies were obtained from Pima Indians (*n* = 69) with type 2 diabetes from the Gila River Indian Community. The study participants were enrolled in a randomised, double-blinded, placebo-controlled interventional clinical trial funded by the National Institute of Diabetes and Digestive and Kidney Diseases (NIDDK) [13]. The study was approved by the Institutional Review Board of the NIDDK and each participant signed an informed consent document. Kidney tissue processing and microarray preparation were described previously [14]. Gene expression profiling and pre-processing were done using GeneChip Human Genome series U133A and Plus 2.0 Array (Affymetrix, Santa Clara, CA, USA) [15, 16]. Further details are listed in the Electronic supplementary material (ESM) Methods.

### Nephroseq

Data from the Nephroseq (www.nephroseq.org, University of Michigan, Ann Arbor, MI, USA) and Nephrocell (http://nephrocell.miktmc.org/) databases were extracted to further examine gene expression in human kidney. *IGFBP* expression data were obtained from the datasets: ‘Woroniecka Diabetes Glom’ [17]; ‘Lindenmeyer Normal Tissue Panel’ [18].

### Ex vivo human glomeruli

For ex vivo glomerular studies, glomeruli were isolated from human kidneys that were unsuitable for transplantation. Perfused kidneys were placed on ice and glomeruli were isolated by passing sequentially through sieves with pore sizes 425 μm, 180 μm, 125 μm and 90 μm, as described previously [19]. All studies on human kidney tissue were approved by national and local research ethics committees (Institutional Ethical Committee, South West–Central Bristol National Health Service Research Ethics Committee, UK; and East Midlands–Leicester National Health Service Research Ethics Committee, UK), and conducted in accordance with the tenets of the Declaration of Helsinki.

### Cell culture and stimulations

Conditionally immortalised human podocytes [19] were grown to 80% confluence in RPMI-1640 containing l-glutamine and NaHCO_3_, and supplemented with 10% FBS (Sigma Aldrich, UK), at 33°C with 5% CO_2_ before thermo-switching to 37°C in 5% CO_2_, and allowed to differentiate for 9–12 days. Conditionally immortalised glomerular endothelial cells [20] were maintained in Endothelial Cell Growth Basal Medium-2 (EBM-2), containing microvascular SingleQuots Supplement Pack in 5% FBS (Lonza, UK). All cells were free of mycoplasma contamination. Human podocytes expressing forkhead box O1 (FoxO1)-clover were generated as previously described [21], using pLenti-FoxO1-clover (a gift from P. Rotwein, Addgene plasmid no. 67759; http://n2t/addgene:67759; RRID:Addgene_67759).

Differentiated cells were incubated with serum-, insulin- and IGF-free RPMI-1640, or EBM-2, for 2–6 h, before indicated stimulation. The FoxO1 inhibitor, AS1842856 (Merck, Watford, UK), was used at 50 ng/ml for the stated times. Insulin (100 nmol/l, unless otherwise stated), Wortmannin (phosphoinositide 3-kinase [PI3K] inhibitor, 200 nmol/l) and GSK694002 (Akt inhibitor, 200 nmol/l) were purchased from R&D Systems (Abingdon, UK). Human recombinant IGFBP-1–6 were obtained from Gropep Bioreagents (Australia) and R&D Systems. Doses of IGFBPs were taken from previous publications [9, 22, 23]. Pre-treatment with the anti-β1-integrin antibody, P5D2 (Merck), was at 100 ng/ml or 1000 ng/ml for 30 min.

### Quantitative RT-PCR

Total RNA was isolated using an RNeasy Mini Kit (QIAGEN, Germany), and cDNA was synthesised using a high-capacity RNA-cDNA kit (ThermoFisher Scientific, UK). Quantitative RT-PCR was performed using SYBR green (Sigma Aldrich) in a StepOnePlus system (ThermoFisher Scientific) for human *IGFBP-1* (forward: TTTTACCTGCCAAACTGCAACA, reverse: CCCATTCCAAGGGTAGACGC) normalised to *B-ACTIN* (forward: GACAGGATGCAGAAGGAGATTACT, reverse: TGATCCACATCTGCTGGAAGGT).

### IGFBP-1 ELISA

Cellular and secreted IGFBP-1 levels were quantified using an IGFBP-1 ELISA (R&D Systems), as per the manufacturer’s instructions. For quantification of secreted IGFBP-1 levels, cell-free medium was collected after a 6 or 24 h incubation with cells or glomeruli, 100 μl was added to the ELISA and IGFBP-1 levels were normalised to total protein concentration, quantified using bicinchoninic acid (BCA) (ThermoFisher Scientific) and Bradford assays (Abcam, Cambridge, UK).

### Western blotting

Total protein lysates were extracted using RIPA lysis buffer (Sigma Aldrich), resolved using SDS–PAGE and blotted onto PVDF membranes. Membranes were incubated in primary antibodies overnight at 4°C, before washing and incubation with the appropriate horseradish peroxidase (HRP)-conjugated secondary antibody (Sigma Aldrich) at 1:10,000 dilution. Immunoreactive bands were visualised using Clarity ECL Western Blotting Substrate (Bio-Rad, Hemel Hempstead, UK) on a AI600 imager (GE Healthcare, Amersham, UK) and quantified using ImageJ (NIH, https://imagej.nih.gov/ij/). Primary antibodies are listed in the ESM Methods.

### Active β1-integrin immunoprecipitation

Proteins were extracted in Tris-NaCl-ethylenediaminetetraacetic acid (TNE) (100 mM Tris–HCl [pH 7.4], 150 mM NaCl, 0.1 mM EDTA) containing 2% NP40 and protease inhibitors (Sigma Aldrich). Clarified lysates were incubated with 1 μg of active β1-integrin (Merck) or isotype-matched IgG control, overnight at 4°C, under constant rotation, before incubation with 15 μl of protein-A/G Sepharose for 3 h at 4°C, under rotation. Immune complexes were pelleted at 1000 *g* and washed in chilled TNE. Lysates were resolved on 10% SDS–PAGE gels and probed with a total-β1-integrin antibody (NEB, Hertfordshire, UK).

### Cell motility assay

Cell motility was measured using a modified wound healing assay to model cytoskeletal regulation, as previously described [24]. Cells were grown to confluence in CELLSTAR tissue-culture (TC)-treated six-well culture plates with no additional protein coating; starved of serum, insulin and IGF; and wounded with a sterile 0.2 mm tip. Images were acquired at 0 and 14 h, following stimulation with IGFBPs, and the distance of migration was measured in ImageJ.

### InnoCyte cell adhesion assay

InnoCyte extracellular matrix (ECM) cell adhesion assays (Merck) were performed according to the manufacturer’s instructions. Briefly, differentiated podocytes were resuspended in serum-free RPMI at densities between 100,000 and 500,000 cells/ml. Then, 100 μl of the cell suspension was added to wells of the ECM protein-coated plate and incubated for 2 h at 37°C. The cell suspension was then discarded, and the plate washed with PBS before the addition of a calcein-AM solution and further incubation at 37°C for 1 h. The fluorescence in each well was measured at an excitation wavelength of ~485 nm and an emission wavelength of ~520 nm.

### Puromycin cell survival studies

The Promega CellTiter 96 AQueous One Solution Cell Proliferation Assay (3-(4,5-dimethylthiazol-2-yl)-5-(3-carboxymethoxyphenyl)-2-(4-sulfophenyl)-2H-tetrazolium; MTS assay) was utilised to examine the number of living cells after treatment. Puromycin dihydrochloride (Sigma Aldrich) was used at 50 μg/ml for 18 h to induce cell death.

### Electrical Cell-substrate Impedance Sensing

Resistance measurement was performed using an automated cell monitoring system, electrical cell-substrate impedance sensing (ECIS) (ECIS 1600R, Applied Biophysics, NY, USA), as previously described [25]. Briefly, human podocytes were seeded onto gold microelectrode ECIS arrays (Applied Biophysics) at a density of 1×10^6^ cells/cm^2^. Following differentiation, podocytes were serum-starved for 4 h and stimulated with IGFBP-1–6 or vehicle. Resistance was measured (ohms) at regular intervals for 24 h and presented as a ratio to vehicle-treated cells.

### Semi-automated immunofluorescent imaging and analysis

Podocytes were grown in 96-well plates (Greiner, Stonehouse, UK), stimulated as indicated before fixation and immunostaining. Image acquisition was automated using an IN Cell Analyzer 2200 (GE Healthcare) imaging platform, with a ×10 or ×20 objective, and quantified using IN Cell Analyzer work station 3.5 software, as previously described [26, 27]. At least three technical replicates were performed within each experiment, with four fields of view per well, yielding data for >600 cells per condition, per experiment. For FoxO1-translocation assays, individual cell measurements were used to calculate the percentage of podocytes positive for nuclear FoxO1, where a positive cell had a ratio of nuclear to cytoplasmic (N:C) FoxO1 fluorescence greater than 1. For quantification of phosphorylated FAK and Paxillin, measurements of fluorescence intensity at focal adhesions were used. Phalloidin staining was used to visualise F-actin, the quantification of which was automated using IN Cell Developer software. Additional details can be found in the ESM Methods.

### Statistics

Data are presented as mean ± SEM unless otherwise stated, with further details of statistical analysis provided in relevant figure legends. Analysis was performed using GraphPad Prism v8 (GraphPad Software, CA, USA), where statistical significance was calculated with one-way ANOVA with Tukey’s multiple comparison test or *t* tests. For Nephroseq data, statistical significance is presented as *p* values computed using Welch’s *t* test and *q* values (exported from Nephroseq) corrected using the Benjamini–Hochberg method. For gene expression analysis in the Pima diabetic cohort, differential expression was estimated using R (https://www.r-project.org/) with the limma package [28, 29], comparing control participants and early DKD, and adjusting for age and sex. The *p* values were adjusted using the Benjamini–Hochberg correction.

## Results

### Glomerular *IGFBP-1* expression is reduced in human type 2 DKD

**Fig. 1:**
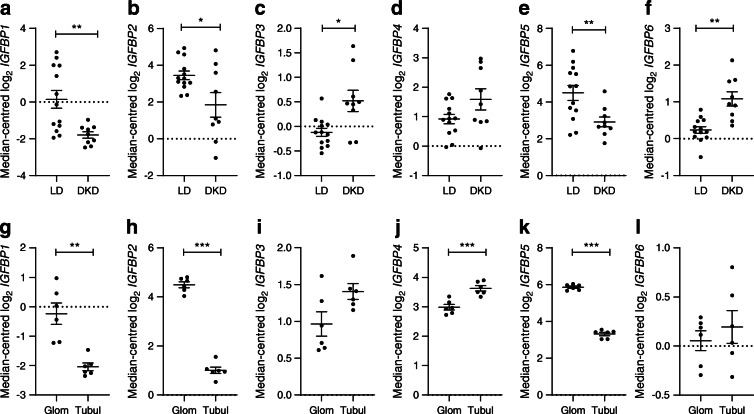
Changes in glomerular IGFBP expression are observed in type 2 DKD. Data extracted from Nephroseq ‘Woroniecka Diabetes Glom’ and ‘Lindenmeyer Normal Tissue Panel’ datasets comparing expression (median-centred log_2_) of (**a**) *IGFBP1* (*q* = 0.02), (**b**) *IGFBP2* (*q* = 0.18), (**c**) *IGFBP3* (*q* = 0.04), (**d**) *IGFBP4* (*q* = 0.41), (**e**) *IGFBP5* (*q* = 0.04) and (**f**) *IGFBP6* (*q* = 0.09), in glomeruli from DKD (*n* = 9) vs healthy living donors (*n* = 13), *p < 0.05, **p < 0.01, Welch’s *t* test, *q* values adjusted using Benjamini–Hochberg method; and (**g**) *IGFBP1* (*q* = 0.006), (**h**) *IGFBP2* (*q* = 1.23×10^−7^), (**i**) *IGFBP3* (*q* = 0.06), (**j**) *IGFBP4* (*q* = 0.001), (**k**) *IGFBP5* (*q* = 1.19×10^−7^) and (**l**) *IGFBP6* (*q* = 0.46) in glomeruli vs tubulointerstitium, *n* = 6 each group, **p < 0.01, ***p < 0.001, Welch’s *t* test, *q* values adjusted using Benjamini–Hochberg method. LD, living donor; Glom, glomeruli; Tubul, tubulointerstitium

To determine whether any differences in local glomerular expression of IGFBPs occurred in DKD, we initially analysed existing data from the renal transcriptomics database Nephroseq. In the ‘Woroniecka Diabetes Glom’ dataset [17], *IGFBP-1*, *-2* and *-5* were downregulated in the glomeruli of diabetic individuals, with *IGFBP-1* being the most highly downregulated gene (−3.83 fold change, *p* = 9.6 × 10^−4^) (Fig. 1a–f). Transcriptome data from normal human kidney [18] indicated that *IGFBP-1*, *IGFBP-2* and *IGFBP-5* were enriched in glomeruli in comparison with the tubulointerstitium (Fig. 1g–l).

We further analysed *IGFBP* expression in the Pima DKD cohort [13], where we also found a reduction in glomerular *IGFBP-1* in individuals with early-stage disease, compared with living donors (Table 1), which, notably, is unlikely to be purely a consequence of podocyte loss in this cohort. *IGFBP-4* was also significantly reduced in the glomeruli of this cohort.

**Table 1:** Glomerular *IGFBP-1* is reduced early in type 2 DKD

Symbol	Early DKD (*n* = 69) vs LD (*n* = 18)		
	FC	log FC	Adj. *p* value
*IGFBP1*	0.67	−0.58	0.04
*IGFBP2*	1.31	0.39	0.00
*IGFBP3*	1.13	0.18	0.53
*IGFBP4*	0.85	−0.24	0.02
*IGFBP5*	0.87	−0.21	0.09
*IGFBP6*	1.39	0.48	0.02

Glomerular expression (log_2_ mRNA intensity) of IGFBPs in the Pima type 2 diabetes cohort with early DKD (*n* = 69) vs healthy living donors (*n* = 18)

Differential expression was estimated using limma, adjusting for age and sex

*p* values were adjusted for multiple testing using the Benjamini–Hochberg correction

Adj., adjusted; FC, fold change; LD, living donor

Collectively, these results demonstrate a local production and control of IGFBPs in the kidney in type 2 DKD, with a consistent reduction in glomerular *IGFBP-1* observed in both diabetic cohorts.

### PI3K–Akt–FoxO1 signalling regulates podocyte *IGFBP-1* expression

Given that we observed a consistent reduction in glomerular *IGFBP-1* expression in both diabetic cohorts, we further explored the mechanisms behind IGFBP-1 regulation. Analysis of single-cell kidney transcriptome data [30] and conditionally immortalised human glomerular cells demonstrated that IGFBP-1 expression and protein secretion were most prominent in podocytes, with some signal in glomerular endothelial cells (ESM Fig. 1).

**Fig. 2:**
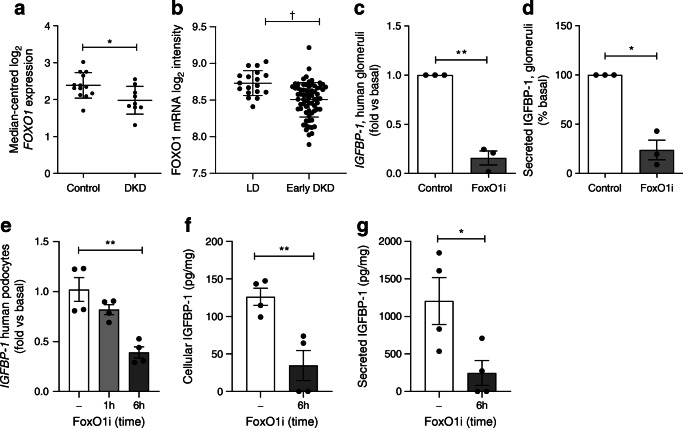
IGFBP-1 expression is controlled by FoxO1 in human glomeruli and podocytes. (**a**) Nephroseq: *FOXO1* expression in the ‘Woroniecka Diabetes Glom’ dataset, healthy living donor (*n* = 13) vs DKD (*n* = 9), *p = 0.02, Welch’s *t* test (*q* value = 0.10). (**b**) Expression (log_2_ mRNA intensity) of *FOXO1* in the Pima type 2 diabetes cohort with early DKD (Early DKD, *n* = 69) vs living donors (LD, *n* = 18), ^†^*q* = 0.0003. (**c**) qPCR results of *IGFBP-1* mRNA in ex vivo human glomeruli and (**d**) IGFBP-1 concentration in glomerular media after FoxO1 inhibition (50 ng/ml AS1842856 FoxO1 inhibitor) for 10 days, *n* = 3 individuals, *p < 0.05, **p < 0.01, *t* test. (**e**) qPCR results of *IGFBP-1* mRNA expression (*n* = 4), **p < 0.01 one-way ANOVA with Tukey’s multiple comparison test. (**f**) IGFBP-1 levels in podocyte lysates normalised to total protein (*n* = 4), **p = 0.007, *t* test. (**g**) IGFBP-1 concentration in cell-free podocyte media following 6 h FoxO1 inhibition (50 ng/ml AS1842856) (*n* = 4), *p = 0.03, *t* test, 6 h vs basal. FoxO1i, FoxO1 inhibitor; qPCR, quantitative PCR

As *IGFBP-1* expression is regulated by activity of the transcription factor FoxO1 in other cell types [31], which is in turn regulated by PI3K–Akt signalling [32], we hypothesised that insulin–PI3K–Akt signalling would control FoxO1-driven *IGFBP-1* expression in glomeruli. To first determine the involvement of FoxO1 in glomerular *IGFBP-1* expression, we studied human glomeruli ex vivo alongside glomerular transcriptomics data from diabetic individuals and conditionally immortalised cell lines. In normal human kidney [18] *FOXO1* was expressed in both glomerular and tubular regions (ESM Fig. 2). In glomeruli of individuals with type 2 DKD [17], we observed a reduction in *FOXO1* expression (Fig. 2a) along with consistent regulation of other reported FoxO1-target genes [33] (ESM Fig. 3), indicating that glomerular FoxO1 is negatively regulated in type 2 DKD. Analysis of the Pima DKD cohort [13] indicated that *FOXO1* suppression occurred early in DKD progression (Fig. 2b). In ex vivo human glomeruli, the inhibition of FoxO1 (with AS1842856) caused a consistent decrease in *IGFBP-1* mRNA (Fig. 2c) and reduced IGFBP-1 protein secretion (Fig. 2d) in each of the three different human glomerular fractions studied. Conditionally immortalised human podocytes also displayed a reduction in *IGFBP-1* mRNA expression after 6 h of FoxO1 inhibition (Fig. 2e), with a corresponding reduction in cellular (Fig. 2f) and secreted (Fig. 2g) IGFBP-1 protein. In contrast, in glomerular endothelial cells, FoxO1 inhibition had no effect on *IGFBP-1* levels (ESM Fig. 4), which may suggest another mechanism of regulation in these cells, such as the mammalian target of rapamycin (mTOR)-dependent (FoxO-independent) regulation of IGFBP-1 which has been described by others [34]. Under basal conditions, FoxO1 overexpression had no effect on *IGFBP-1* levels in podocytes (ESM Fig. 5), suggesting that it is not solely the expression levels of FoxO1 that are important in regulating gene transcription. Treatment with AS1842856 had no effect on podocyte number over the course of the experiment, indicating there were no toxic effects of this compound (ESM Fig. 6).

**Fig. 3:**
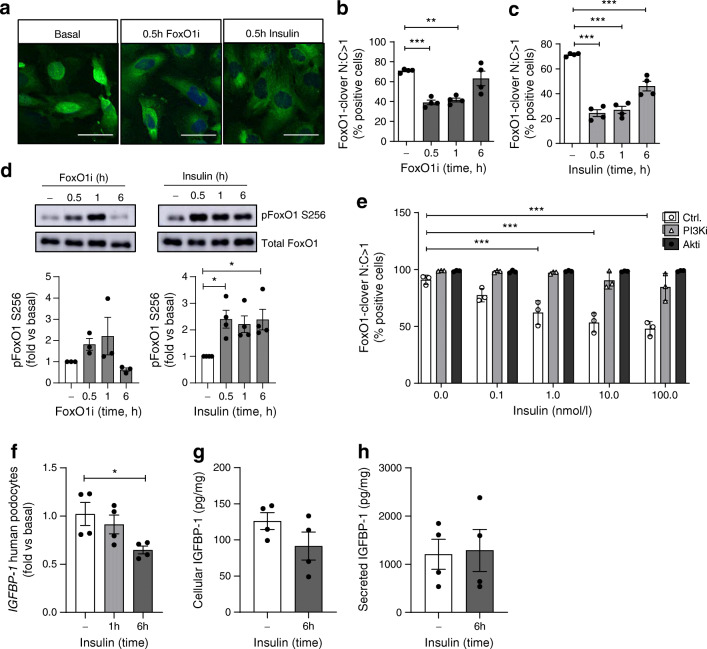
Insulin–PI3K–Akt signalling regulates FoxO1 activity and IGFBP-1 expression in human podocytes. (**a**) Representative images (scale bar, 50 μm) of human podocytes stably expressing FoxO1-clover and quantification of nuclear FoxO1-clover levels following (**b**) FoxO1 inhibition (50 ng/ml AS1842856) or (**c**) insulin stimulation (100 nmol/l), *n* = 4 experiments, **p < 0.01, ***p < 0.001, one-way ANOVA with Tukey’s multiple comparison test. (**d**) Representative western blots and matched densitometry demonstrating an increase in phosphorylation of FoxO1 (Ser 256) following insulin stimulation (100 nmol/l), *n* = 4, *p < 0.05 at 0.5 h, 1 h and 6 h, one-way ANOVA with Tukey’s multiple comparison test. (**e**) Quantification of nuclear FoxO1-clover in podocytes following 30 min insulin stimulation at the stated doses, with or without additional inhibition of PI3K (200 nmol/l wortmannin) or Akt (200 nmol/l GSK694002), *n* = 3, ***p < 0.001, one-way ANOVA with Tukey’s multiple comparison test. (**f**) qPCR results of *IGFBP-1* mRNA following insulin stimulation (100 nmol/l), *p < 0.05, one-way ANOVA with Tukey’s multiple comparison test, *n* = 4. (**g**) IGFBP-1 levels (pg) to total protein (mg) in podocyte lysates, *p* = 0.17, unpaired *t* test, and (**h**) cell-free podocyte media, following insulin stimulation (100 nmol/l), *p* = 0.88, unpaired *t* test, *n* = 4. Akti, Akt inhibitor; Ctrl., control; FoxO1i, FoxO1 inhibitor; N:C, ratio of nuclear to cytoplasmic; PI3Ki, PI3K inhibitor

Investigating the role of insulin–PI3K–Akt signalling in this pathway, in FoxO1-clover reporter podocytes [21] both FoxO1 inhibition with AS1842856 and insulin stimulation reduced the nuclear levels of FoxO1 (Fig. 3a–c) and increased FoxO1 phosphorylation (Fig. 3d). In the presence of PI3K or Akt inhibitors, the dose-dependent effect of insulin on nuclear FoxO1 exclusion was abolished (Fig. 3e), indicating that insulin–PI3K–Akt signalling inhibits FoxO1 nuclear localisation and activity in human podocytes. Furthermore, insulin stimulation alone reduced cellular *IGFBP-1* mRNA (Fig. 3f) in podocytes at 6 h, although we did not detect any significant changes in IGFBP-1 protein by insulin at this time (Fig. 3g, h), which may be due to assay sensitivity or sensitivity of other necessary co-factors to insulin stimulation within the time frame. The effects of insulin on *IGFBP-1* mRNA expression were also abolished when PI3K was inhibited (ESM Fig. 7).

Thus, increased insulin–PI3K–Akt signalling inhibits FoxO1 transcriptional activity in human podocytes and FoxO1 inhibition leads to suppression of *IGFBP-1* in podocytes and in human glomeruli.

### IGFBPs influence podocyte function

Podocyte function at the filtration barrier depends on cell adhesion to the GBM and co-ordinated regulation of foot process dynamics, largely by control of the actin cytoskeleton. To investigate whether IGFBP-1 could have effects on these responses we performed ECIS, migration, adhesion and survival assays on conditionally immortalised human podocytes that were starved of serum and IGF and stimulated with IGFBPs.

**Fig. 4:**
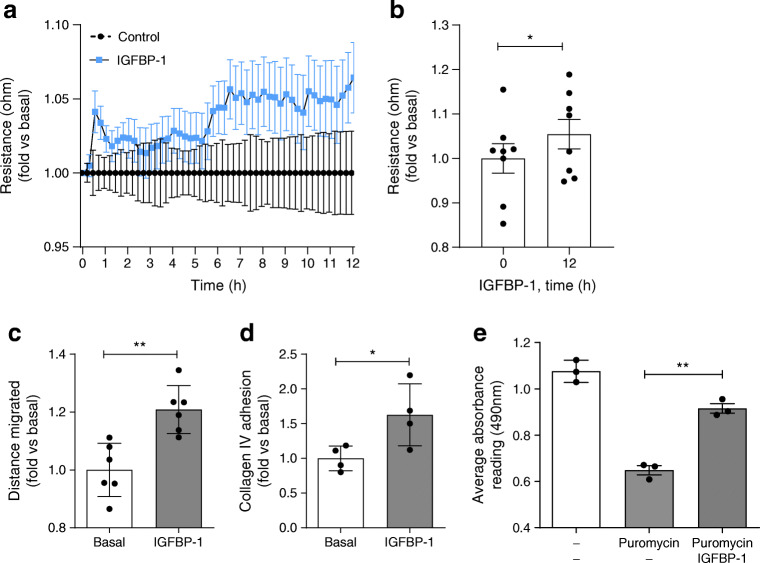
Stimulation of human podocytes with IGFBP-1 influences cellular function. Human podocytes were starved of serum (including IGF) for 2–4 h before IGFBP-1 stimulation (5 nmol/l). (**a**) ECIS analysis over 12 h suggesting increased resistance across adherent human podocytes following IGFBP-1 treatment, normalised to time 0 and vehicle-treated cells, repeated measures one-way ANOVA, *p* = 0.11. (**b**) Bar chart of ECIS analysis, normalised to mean at time 0, demonstrating an increase in resistance 12 h post IGFBP-1 stimulation, *p = 0.05, paired *t* test, *n* = 8. (**c**) Human podocytes were stimulated with IGFBP-1 and motility was assessed over a period of 12–14 h and normalised to the mean of the basal (unstimulated) conditions, **p = 0.002, unpaired *t* test, *n* = 6. (**d**) Change in podocyte adhesion to collagen IV following IGFBP-1 stimulation, *n* = 4, *p = 0.04, unpaired *t* test. (**e**) Cell viability after exposure of podocytes to puromycin. Human podocytes were treated with puromycin (50 μg/ml, 18 h) with or without IGFBP-1 treatment. A significant increase in the number of viable cells was apparent with IGFBP-1 treatment, determined by MTS assays, **p < 0.01, *n* = 3, one-way ANOVA with Tukey’s multiple comparison test

Measuring electrical resistance across adherent podocytes in vitro, which indicates changes in cell–cell junctions or cellular adhesion [25], we found an increase in electrical resistance following treatment with IGFBP-1, which was significant at 12 h (Fig. 4a, b). We also observed increased resistance following stimulation with IGFBP-2, IGFBP-3 and IGFBP-4 (ESM Fig. 8), indicating that several IGFBPs may have direct effects on podocyte structure and function.

Studying podocyte motility (as an indication of cytoskeletal signalling and foot process dynamics in vitro), we observed an increase in the migratory response in cells stimulated with IGFBP-1 (Fig. 4c, ESM Fig. 9a), suggesting an increase in cytoskeletal organisation. Using protein-specific adhesion assays, we also observed an increase in podocyte adhesion to type IV collagen, a major component of the mature GBM [35], following stimulation with IGFBP-1 (Fig. 4d) and IGFBP-4 (ESM Fig. 9b), suggesting that these IGFBPs may improve podocyte attachment to the GBM. Finally, we assessed the direct role of IGFBPs in podocyte survival. Following pre-treatment with puromycin, we found IGFBP-1 stimulation significantly increased podocyte viability, measured using MTS assays (Fig. 4e).

Collectively, these observations indicate that IGFBP-1 can influence a variety of podocyte functional responses in vitro*.* We found IGFBP-2–6 also had effects on podocytes and, as such, further study of other IGFBPs in this context is warranted in the future.

### IGFBP-1 regulates β1-integrin and focal adhesion signalling in podocytes

**Fig. 5:**
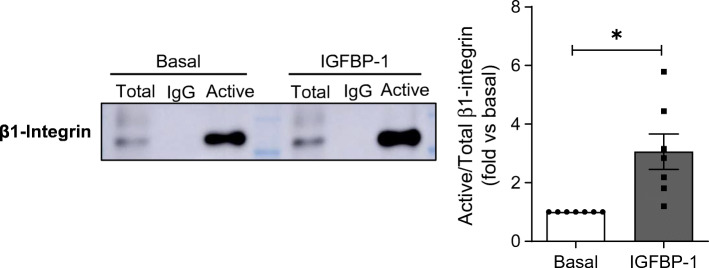
IGFBP-1 stimulation increases β1-integrin activation in human podocytes. Human podocytes were stimulated with IGFBP-1 (50 ng/ml, 15 min) before immunoprecipitation with antibodies specifically recognising conformationally active β1-integrin. Representative western blot for β1-integrin in both total cell lysates (‘Total’) and immunoprecipitated fractions (‘Active’), and densitometry representing the ratios of Active to Total β1-integrin, indicating a higher proportion of Active β1-integrin following IGFBP-1 stimulation, *n* = 7, *p < 0.05, *t* test

Given that we found IGFBP-1 influenced multiple processes in human podocytes, we also further explored IGFBP-1-mediated podocyte signalling, focusing on signalling responsible for controlling cell adhesion/migration and the actin cytoskeleton. Interestingly, IGFBP-1 contains functional integrin-binding Arg-Gly-Asp (RGD) domains, and is thought to exert its IGF-independent actions via β1-integrin signalling [8, 10]. As β1-integrins are central in controlling cellular adhesion and migration, we investigated whether IGFBP-1 influenced β1-integrin activity in podocytes. Using antibodies recognising the active (‘extended’) conformation of β1-integrin, we found a higher proportion of active β1-integrin following IGFBP-1 treatment, when compared with basal cells (Fig. 5).

**Fig. 6:**
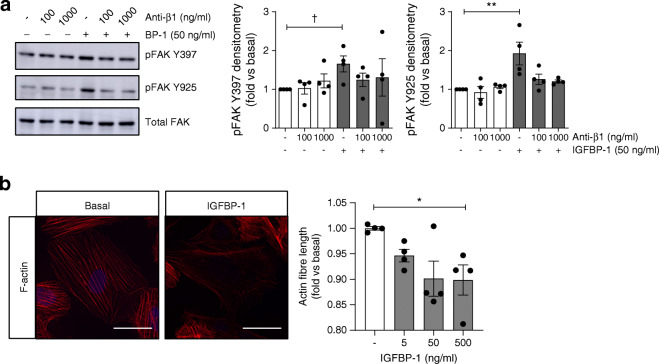
IGFBP-1 stimulates FA signalling in human podocytes. (**a**) Representative western blots and densitometry demonstrating the phosphorylation of FAK Y397 and Y925 in human podocytes stimulated with 50 ng/ml IGFBP-1 for 15 min, with or without pre-treatment with anti-β1-integrin (Anti-β1), P5D2 (100 ng/ml or 1000 ng/ml, 30 min), ^†^*p* < 0.05, *t* test vs unstimulated, **p = 0.004, one-way ANOVA with Tukey’s multiple comparison test, *n* = 4. (**b**) Representative images (scale bar, 50 μm) and automated quantification of mean F-actin fibre length per cell in human podocytes stimulated with IGFBP-1 at the stated concentrations for 60 min (*n* = 4, 3 replicates per condition, *p = 0.04, one-way ANOVA with Tukey’s multiple comparison test, 500 ng/ml vs basal). Modest changes in brightness and contrast were uniformly applied to all images for visualisation; unmodified images were used for quantification

Protein complexes termed focal adhesions (FAs) link integrins to the actin cytoskeleton and act as mediators of integrin signalling. In human podocytes stimulated with IGFBP-1, we found an increase in focal-adhesion kinase (FAK) phosphorylation at both the auto-phosphorylation site, Y397, and the Src-regulated phosphorylation site, Y925 (Fig. 6a, ESM Fig. 10a), with no effect on the IGF-IR (phosphorylation at Tyr1135/1136) (ESM Fig. 10b). The pre-treatment of podocytes with the β1-integrin blocking antibody, P5D2 [36, 37], reduced IGFBP-1-stimulated FAK phosphorylation (Fig. 6a), although some variability in the response was observed with higher doses of P5D2. We observed no effects on FAK phosphorylation with P5D2 treatment alone at these concentrations. Using automated imaging and analysis, we found that changes in FAK phosphorylation did not occur within the nucleus (ESM Fig. 11) and that this response occurred alongside a small increase in the phosphorylation of paxillin (Y118) at FAs (ESM Fig. 12a, b).

We used automated imaging and analysis to quantify changes in the actin cytoskeleton and found a reduction in length of F-actin fibres after 60 min of IGFBP-1 treatment (Fig. 6b), indicating an increase in F-actin remodelling, although no significant changes in F-actin structures were observed at the shorter time point (ESM Fig. 13). These results suggest that, similar to other cell systems [9, 38], IGFBP-1 can regulate β1-integrin/FA/F-actin signalling in human podocytes.

## Discussion

IGFBP-1 is a circulating peptide which is often implicated in metabolic homeostasis. Lower levels of IGFBP-1 in the circulation predict type 2 diabetes [39], the metabolic syndrome [40] and the risk of developing cardiovascular disease [41]. Furthermore, disruptions to IGFBP-1 have also been associated with diabetic nephropathy [42], with polymorphisms in IGFBP-1 associated with kidney disease in type 2 diabetes [7].

In the present study, we investigated the local control and actions of IGFBPs in the glomerulus and found that glomerular *IGFBP-1* was significantly reduced in individuals with type 2 DKD, where it was expressed and secreted by podocytes, demonstrating local glomerular control of IGFBP-1. Furthermore, we found IGFBP-1 had direct, IGF-independent effects on podocytes, by increasing podocyte adhesion and migration in vitro as well as increasing electrical resistance across the adhesive cell layer, responses that relate to control of the actin cytoskeleton and are important for cell function at the filtration barrier. It also had pro-survival effects on puromycin-treated podocytes.

Mechanistically, we found that glomerular *IGFBP-1* expression was controlled by FoxO1 activity, which was in turn regulated by insulin–PI3K–Akt signalling in podocytes, consistent with findings in other tissues including the liver, the major source of circulating IGFBP-1 [32]. Indeed, reductions in circulating IGFBP-1 are often attributed to the inhibitory effects of hyperinsulinaemia on hepatic *IGFBP-1* expression, prior to the development of hepatic insulin resistance. As podocytes are insulin-sensitive cells, with both insulin resistance and excessive signalling activity being detrimental to cell function [26, 27, 43], it is plausible that the reduced glomerular *IGFBP-1* observed early in type 2 DKD could also be a consequence of excessive insulin signalling, prior to the development of podocyte insulin resistance, in this regard mirroring the regulation of *IGFBP-1* expression in the liver early in diabetes.

Our study also supports the notion that reduced FoxO1 expression and activity occurs in DKD [44], as we found both a reduction in glomerular *FOXO1* in two diabetic cohorts and consistent regulation of FoxO1-target genes. In contrast to these findings, an increase in *Igfbp-1* has previously been reported in the kidney and liver of *db/db* mice [45], with an apparent decrease in phosphorylated FoxO1. These contradictory results may in part be explained if insulin resistance was apparent in the kidneys of the *db/db* mice at the time point studied, which would plausibly increase FoxO1 activity, similar to the increased *Igfbp-1* expression observed in rodent models of type 1 diabetes [46] which occurs due to an absence of insulin and insulin signalling. Notably, others have previously shown that FoxO1 overexpression is beneficial in DKD, at least in part by the protection of podocytes against epithelial–mesenchymal transition [47]; based on our findings it would be interesting to determine the role of IGFBP-1 in this pathway.

Further exploring the IGF-independent IGFBP-1 signalling responses in podocytes, we found an increase in active β1-integrin following IGFBP-1 treatment. This is in line with other cell systems, where the effects of IGFBP-1 on cellular adhesion, motility and survival have been attributed to the modulation of β1-integrin/FAK [9, 10]. In podocytes, the regulation of β1-integrin is particularly important, as highlighted in podocyte-specific β1-integrin-knockout mice, which develop a severe proteinuric renal disease from birth [48, 49], and in patients where stabilising β1-integrin activation protects against proteinuria [50, 51], implicating an important, IGF-independent role of IGFBP-1 in podocyte health via its ability to modulate β1-integrin activity. This also indicates the mechanism by which IGFBP-1 may influence podocyte motility, adhesion and electrical resistance.

Downstream of β1-integrin, we found that IGFBP-1 stimulated the phosphorylation of FAK, a central component of FAs involved in cell migration, adhesion, spreading and survival [52], which was blocked in the presence an anti-β1-integrin antibody known to suppress β1-integrin function [36, 37]. Furthermore, we observed a reduction in F-actin stress fibres after IGFBP-1 treatment, supporting a role of IGFBP-1 in regulating FA signalling and the podocyte cytoskeleton. Importantly, these responses were demonstrated in the absence of IGF-I and occurred with no change in activation of IGF-IR.

As podocyte function at the filtration barrier depends on adhesion to the GBM, with detachment from the basement membrane being a major driving factor in albuminuria [53], and changes in podocyte motility and cytoskeletal organisation often associated with changes in foot process dynamics in vivo [54], the ability of IGFBP-1 to control these responses has important implications for podocyte function. Indeed, increased FAK activity has been associated with collagen adhesion [55] and pro-survival signalling, relating to F-actin modulation [56], indicating there are beneficial roles of FAK activity in podocyte biology, which mirrors our findings. However, there is also evidence that increased FAK activity occurs in glomerular disease, with podocyte-specific knock-down of FAK protecting against foot process effacement in vivo*,* corresponding with increased stress fibre formation and reduced cell migration in vitro [57]. These differences likely reflect the complexity of FA signalling and the necessity for controlled regulation of these signalling pathways. Whether the activation of FAK is ultimately beneficial or detrimental also likely depends on context and localisation within cells. Additionally, it is increasingly acknowledged that 2D in vitro cell culture conditions have an impact on these pathways. As such, studies on the dynamic regulation of these signalling pathways by IGFBP-1 in vivo will be beneficial in future.

Others have also previously found that transgenic mice overexpressing human IGFBP-1 from the liver developed glomerular disease [58], suggesting persistent increases in systemic IGFBP-1 may be detrimental. Although the mechanisms of this glomerular phenotype were not defined, it is possible that this was a consequence of reduced IGF-I bioavailability during kidney development. There is also evidence that IGFBP-1 may increase in nephrotic syndrome [5, 6]. Further work is clearly required to determine whether increasing (or maintaining) glomerular IGFBP-1 expression is beneficial to podocyte function early in type 2 DKD in addition to defining the IGF-dependent effects of IGFBP-1 in renal cells.

Although the effects of increasing IGFBP-1 in podocytes in type 2 DKD in vivo are to be determined, protective effects of IGFBP-1 in diabetes and insulin resistance have been shown in models of cardiovascular disease, where it can improve blood pressure, NO production, atherosclerosis and vascular repair via its integrin-binding RGD domain [23, 38, 59]. Interestingly, increased circulating IGFBP-1 has also been shown to improve insulin sensitivity of pancreatic beta cells and skeletal muscle, again by increased RGD signalling [23]. It is therefore also possible that a loss of glomerular *IGFBP-1* expression could contribute towards podocyte insulin resistance in type 2 diabetes. Given the importance of regulated insulin signalling in podocytes [26, 43, 60], it would also be interesting to determine whether maintaining IGFBP-1 (RGD) signalling has similar beneficial effects on podocyte insulin sensitivity in this context.

In summary, this study demonstrates that IGFBP-1 can have direct, IGF-independent effects on podocyte function, modulating β1-integrin-FA pathways. We observed a significant reduction in glomerular *IGFBP-1* early in type 2 DKD, where it is expressed by podocytes and regulated by the transcription factor FoxO1. We further confirm the regulation of FoxO1 activity by insulin–PI3K–Akt signalling in podocytes and provide additional evidence that FoxO1 is suppressed in the glomerulus in type 2 DKD. Thus, increased insulin signalling in early diabetes, prior to the development of glomerular insulin resistance, may suppress FoxO1 activity and *IGFBP-1* expression in the glomerulus and associated signalling. Strategies to maintain IGFBP-1 levels in diabetes may support podocyte function and be therapeutically beneficial.

## Supplementary Information

ESM (PDF 601 kb)

## Data Availability

All data generated or analysed during this study are included in this published article (and its supplementary information files) or are available from the corresponding author upon reasonable request.

## Abbreviations

DKD: Diabetic kidney disease
ECIS: Electrical cell-substrate impedance sensing
EBM-2: Endothelial cell growth basal medium-2
ECM: Extracellular matrix
FA: Focal adhesion
FAK: Focal-adhesion kinase
FoxO1: Forkhead box O1
GBM: Glomerular basement membrane
IGFBP: IGF binding protein
IGF-IR: IGF-I receptor
MTS: 3-(4,5-Dimethylthiazol-2-yl)-5-(3-carboxymethoxyphenyl)-2-(4-sulfophenyl)-2H-tetrazolium
NIDDK: National Institute of Diabetes and Digestive and Kidney Diseases
PI3K: Phosphoinositide 3-kinase
TNE: Tris-NaCl-ethylenediaminetetraacetic acid
