# Supplementary material for: IGFBP-1 expression is reduced in human type 2 diabetic glomeruli and modulates β1-integrin/FAK signalling in human podocytes
Source: Diabetologia. 2021 Mar 24;64(7):1690–702. doi: 10.1007/s00125-021-05427-1 (PMC8187213; doi:10.1007/s00125-021-05427-1)
Supplement: Supplementary file 1 — (PDF 601 kb) [file 125_2021_5427_MOESM1_ESM.pdf]

Supplementary Material

ESM Methods

Human samples

Protocol human kidney biopsies were obtained from Pima individuals (n=69) with type 2 diabetes from the Gila River Indian Community. The study participants were enrolled in a randomized, double-blinded, placebo-controlled interventional clinical trial ("Renoprotection in Early Diabetic Nephropathy in Pima Indians" clinical trial OH95-DK-N037; ClincalTrials.gov number NCT00340678) funded by the National Institute of Diabetes and Digestive and Kidney Diseases (NIDDK) [1]. The study was approved by the Institutional Review Board of NIDDK and each participant signed an informed consent document. All the study participants had normal to microalbuminuria at the time of enrolment. Kidney tissue processing and microarray preparation were described previously [2]. Gene expression profiling and pre-processing were done using GeneChip Human Genome series U133A and Plus 2.0 Array (Affymetrix, Santa Clara, CA) [3, 4]. Owing to ethical considerations, privacy protection, and to avoid identifying individual study participants in this vulnerable population, the Institutional Review Board of the National Institute of Diabetes and Digestive and Kidney Diseases has stipulated that individual-level gene expression and genotype data from this study cannot be made publicly available [4].

Western blotting antibodies

Primary antibodies against Phospho-FoxO1 (Ser256), Total FoxO1, Phospho-IGF-IRβ (Tyr1135/1136)/IRβ (Tyr1150/1151), Total IGF-IRβ, Phospho-FAK (Y397, Y925), Total FAK, Total β1-integrin were purchased from Cell Signalling technologies and diluted 1:1000. β-actin was purchased from Sigma and diluted 1:10,000.

Semi-automated immunofluorescent imaging and analysis

For automated imaging and analysis, podocytes were grown in 96-well plates (Greiner), stimulated as indicated before fixation in 4% PFA. Cells were permeabilised in 0.1% Triton-X100, stained overnight with primary antibodies and for 2 hours with appropriate alexafluor-labelled secondary antibodies and/or alexafluor phalloidin 647 (Invitrogen). Nuclear regions were defined by Hoechst 33342 stains. Image acquisition was automated using an IN Cell Analyzer 2200 (GE Healthcare) high content imaging platform with a 10x or 20x objective. Quantification of IN Cell images was performed using the IN Cell Analyser work station 3.5 software. 3 technical replicates were performed within each experiment, with 4 fields of view per well; yielding data for >600 cells per condition, per experiment. For FoxO1 translocation assays, the intensity of FoxO1-clover was measured in cytoplasmic (defined as a 3µm nuclear collar) and nuclear regions. The ratio of nuclear to cytoplasmic (N:C) fluorescence was calculated for individual cells (at least 2000 cells per condition, per experiment), where N:C ratios of >1 defined cells positive for nuclear FoxO1 localisation. For quantification of phospho-FAK and phospho-Paxillin staining, cells were segmented based on Hoechst and phalloidin staining and fluorescence intensity was measured at Focal adhesions and presented as a ratio to basal conditions. For F-actin quantification, the average length of F-actin filaments per cell was calculated using developer software.

ESM Figures

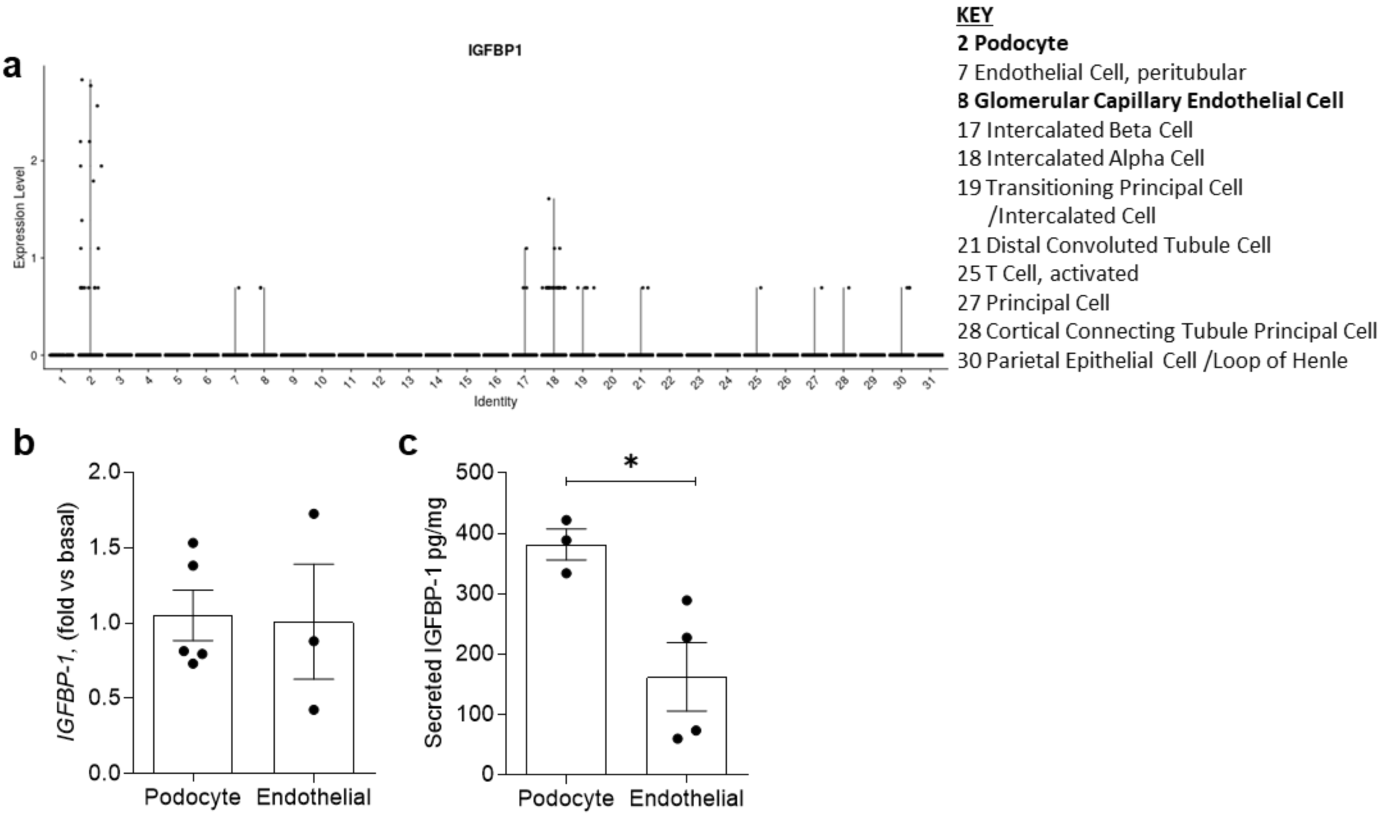

ESM Fig. 1: IGFBP-1 expression in the glomerulus

(a) Nephrocell: Single-cell RNAseq analysis indicating *IGFBP-1* expression in human podocytes, n=24 kidneys; (b) *IGFBP-1* mRNA expression in human conditionally immortalised podocytes (n=5) and glomerular endothelial cells (n=3) determined by qPCR and (c) IGFBP-1 ELISA concentration in cell-free culture media after 24 hours, in human conditionally-immortalised podocytes (n=3) and glomerular endothelial cells (n=4), normalised to total protein; *p=0.03, unpaired t-test.

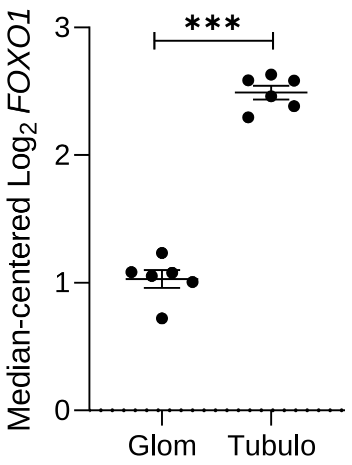

ESM Fig. 2: *FOXO1* is expressed in both glomerular and tubular regions in normal human kidney

Nephroseq: *FOXO1* expression in Lindenmeyer Normal Tissue Panel, Tubulointerstitium vs. Glomeruli Tissue n=6 each group ***P<0.001, Welch’s t-test

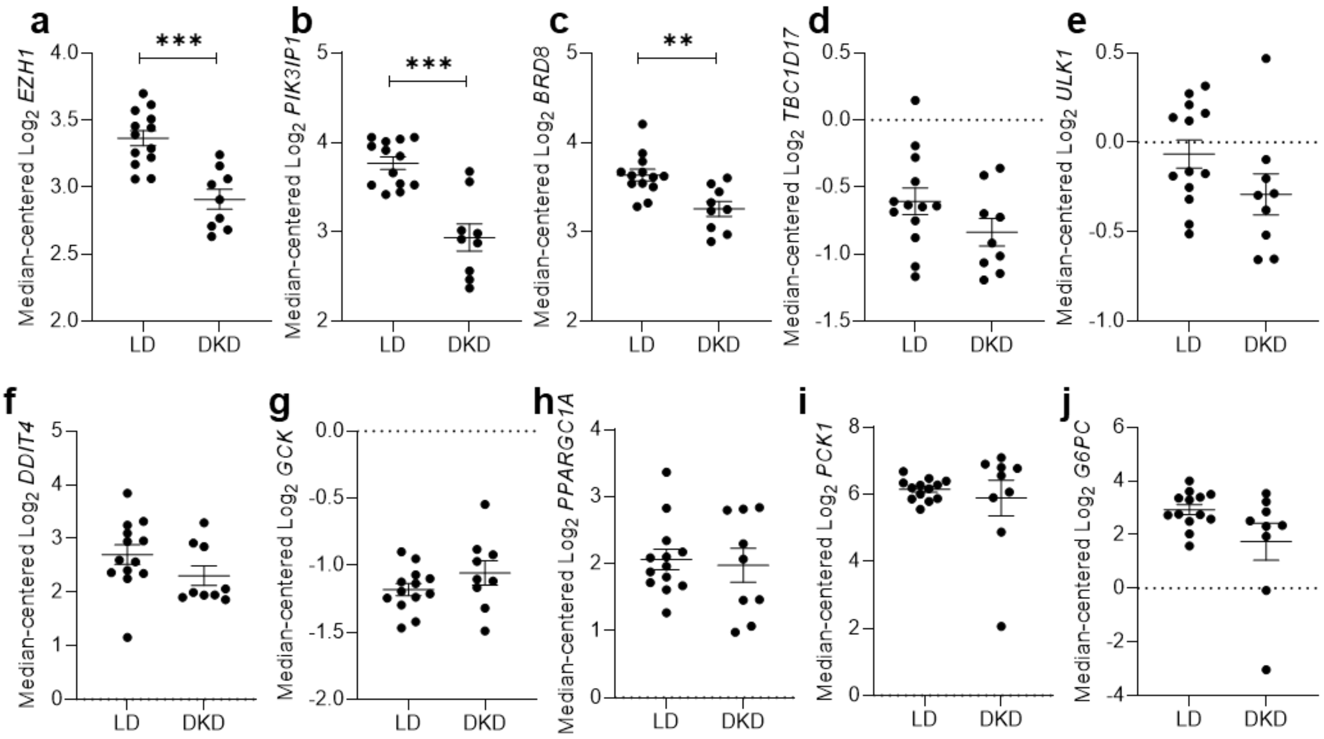

ESM Fig. 3: Glomerular expression of FoxO1-target genes in human diabetic kidney disease

Data extracted from the Nephroseq database "Woroniecka Diabetes Glom" dataset demonstrating expression (median-centered log2) of FoxO1-target genes, described by Batista et al. supporting glomerular FoxO1 inhibition; (a) *EZH1* (q=0.003), (b) *PIK3IP1* (q=0.005), (c) *BRD8* (q=0.02), (d) *TBC1D17* (q=0.34, p=0.06), (e) *ULK1* (q=0.34, p=0.06), (f) *DDIT4* (q=0.37, p=0.07), (g) *GCK* (q=0.56, p=0.12), (h) *PPARGC1A* (q=0.93, p=0.39), (i) *PCK1* (q=0.86, p=0.33), (j) *G6PC* (q=0.34, p=0.13), Diabetic Kidney Disease (n=9) vs. Healthy Living Donor (n=13), **p<0.01, ***p<0.001 Welch's t-test, and FDR-adjusted q-values as exported from the nephroseq database.

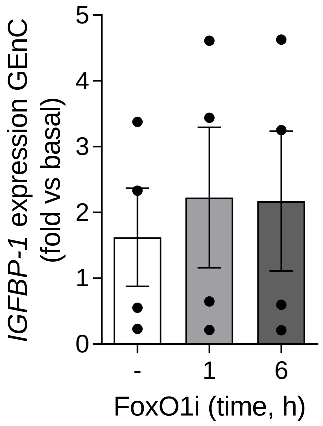

ESM Fig. 4: FoxO1 inhibition has no effect on *IGFBP-1* expression in glomerular endothelial cells

Conditionally-immortalised glomerular endothelial cells (GEnC) were treated with 50ng/ml AS1842856 FoxO1-inhibitor (FoxO1i), *IGFBP-1* mRNA expression was determined by qPCR (n=4, p=0.92, One-way ANOVA, Tukey's multiple comparison)

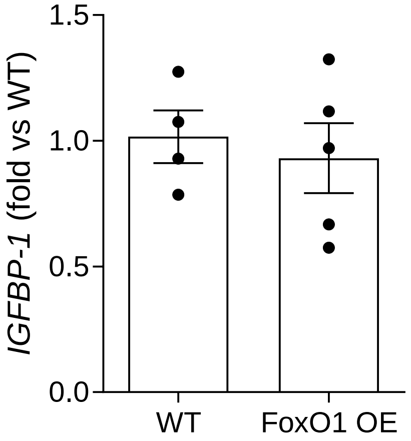

ESM Fig. 5: Podocyte FoxO1-overexpression has no effect on *IGFBP-1* under basal conditions

qPCR results showing *IGFBP-1* expression in conditionally-immortalised human podocytes (WT, n=4) and FoxO1-overexpressing (FoxO1 OE, n=5) podocytes under basal conditions, showing no significant differences in expression, p=0.65, unpaired t-test.

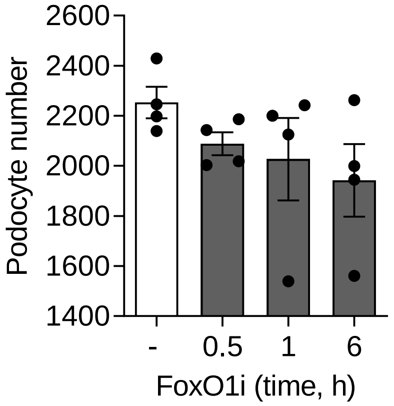

ESM Fig. 6: FoxO1 inhibition does not influence podocyte number

Automated quantification of podocyte number (Hoechst stained cells), shows no significant difference following FoxO1 inhibition (FoxO1i) with 50ng/ml AS1842856, n=4, p=0.28, One-Way ANOVA and Tukey’s multiple comparison.

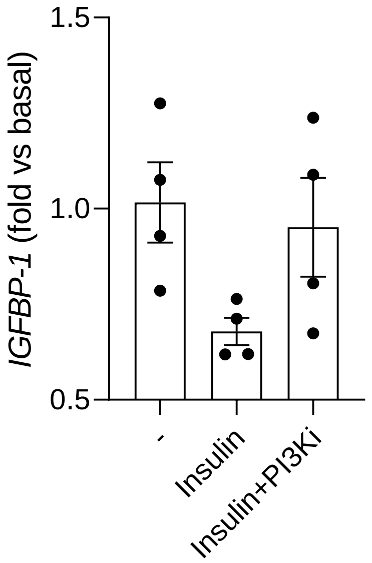

ESM Fig. 7: *IGFBP-1* expression is regulated by Insulin-PI3K signalling in human podocytes

qPCR results showing *IGFBP-1* expression in conditionally-immortalised human podocytes after treatment with insulin (100nmol/l, 6h), with or without PI3K inhibition with wortmannin (200nmol/l, 6h), n=4, One-Way ANOVA with Tukey's multiple comparison test p=0.17, unadjusted p=0.08.

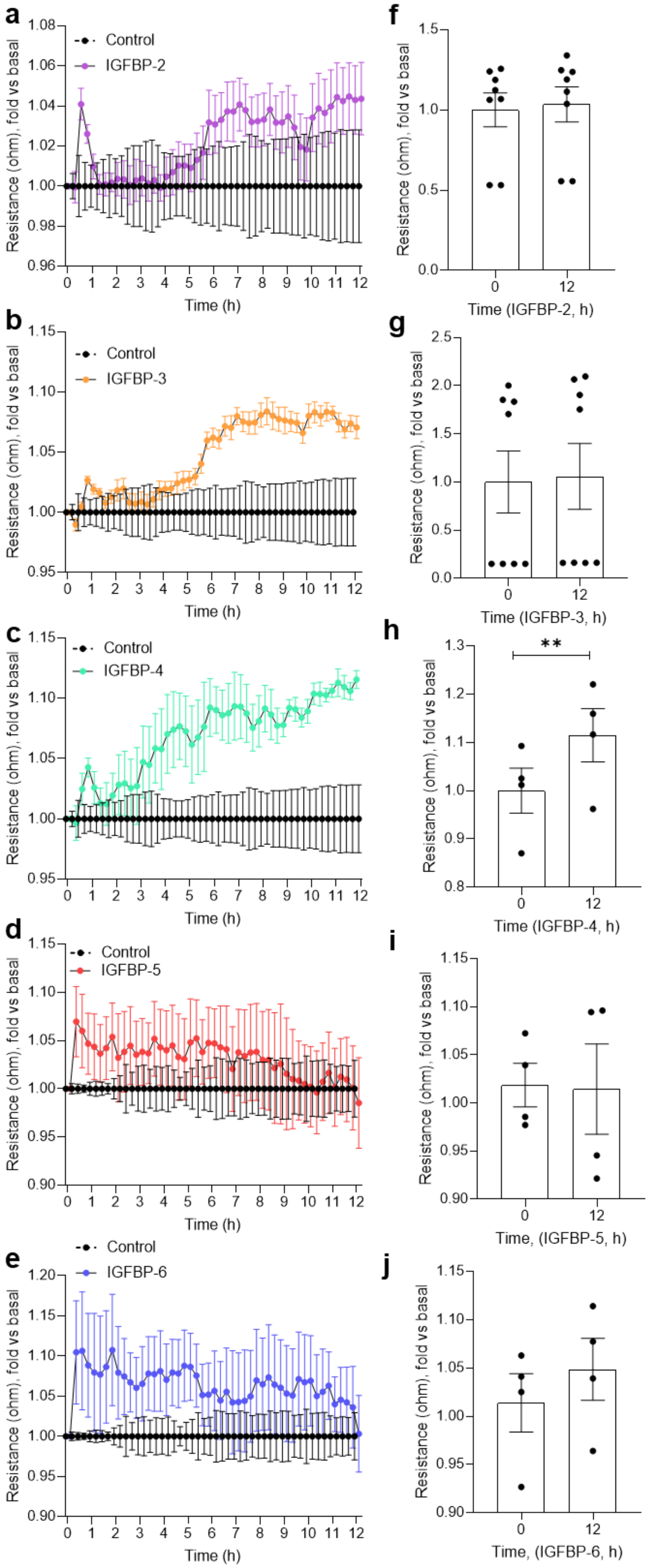

a
Control
IGFBP-2
Resistance (ohm), fold vs basal
Time (h)
b
Control
IGFBP-3
Resistance (ohm), fold vs basal
Time (h)
c
Control
IGFBP-4
Resistance (ohm), fold vs basal
Time (h)
d
Control
IGFBP-5
Resistance (ohm), fold vs basal
Time (h)
e
Control
IGFBP-6
Resistance (ohm), fold vs basal
Time (h)
f
Resistance (ohm), fold vs basal
Time (IGFBP-2, h)
g
Resistance (ohm), fold vs basal
Time (IGFBP-3, h)
h
**
Resistance (ohm), fold vs basal
Time (IGFBP-4, h)
i
Resistance (ohm), fold vs basal
Time, (IGFBP-5, h)
j
Resistance (ohm), fold vs basal
Time, (IGFBP-6, h)

ESM Fig. 8: Podocyte ECIS analysis following stimulation with IGFBPs

Human podocytes were starved of serum (including IGF) for 2-4 hours before IGFBP stimulation at the following concentrations: IGFBP-2 20nmol/l, n=8; IGFBP-3 100nmol/l, n=8; IGFBP-4 10nmol/l, n=4; IGFBP-5 15nmol/l, n=4; IGFBP-6 20nmol/l, n=4. (a-e) Electric Cell Impedance Sensing (ECIS) analysis over 12 hours indicating changes in resistance across adherent human podocytes, normalised to vehicle-treated cells and time 0, following treatment with (a) IGFBP-2, RM One-way ANOVA p=0.02, (b) IGFBP-3 RM One-way ANOVA p<0.0001, (c) IGFBP-4 RM One-way ANOVA p=0.06, (d) IGFPB-5 RM One-way ANOVA p=0.059, (e) IGFBP-6 RM One-way ANOVA p=0.035 and (f-j) bar charts to demonstrate resistance analysis 12 hours post-stimulation, normalised to the average response at time 0, with statistics computed using paired t-tests for (f) IGFBP-2, p=0.15 (g) IGFBP-3, p=0.06 (h) IGFBP-4, **p=0.001 (i) IGFBP-5, p=0.94 (j) IGFBP-6, p=0.48.

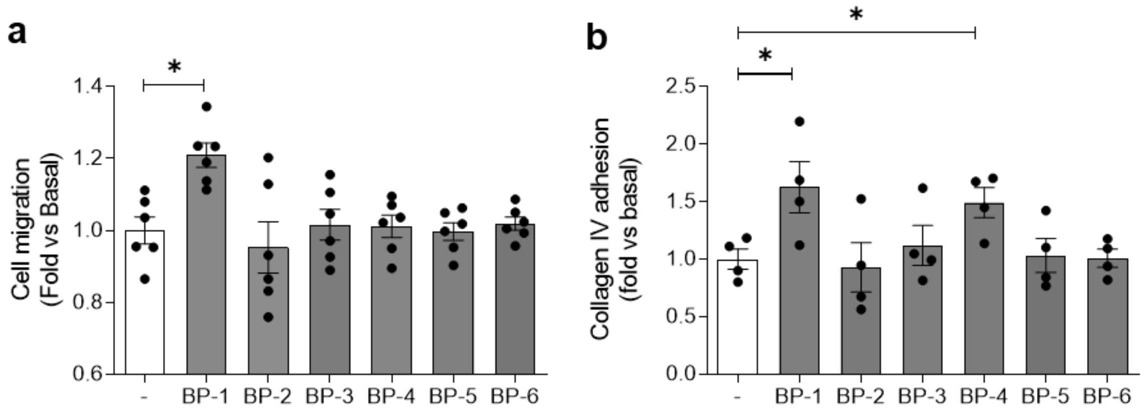

ESM Fig. 9: IGFBPs influence on podocyte adhesion and migration

Human podocytes were starved of serum (including IGF) for 2-4 hours before IGFBP stimulation at the following concentrations: IGFBP-1 5nmol/l, IGFBP-2 20nmol/l, IGFBP-3 100nmol/l, IGFBP-4 10nmol/l, IGFBP-5 15nmol/l, IGFBP-6 20nmol/l. (a) Podocyte motility was assessed over a period of 12-14 hours. Each condition is expressed as a ratio to the average of the basal (unstimulated) response, *p=0.013, One-way ANOVA with Tukey's multiple comparisons test, n=6; (b) Podocyte adhesion to type IV collagen following IGFBP treatment, vs the average of the basal (unstimulated) response, *p=0.04 IGFBP-1 and *p=0.02 IGFBP-4 vs basal, t-test, n=4.

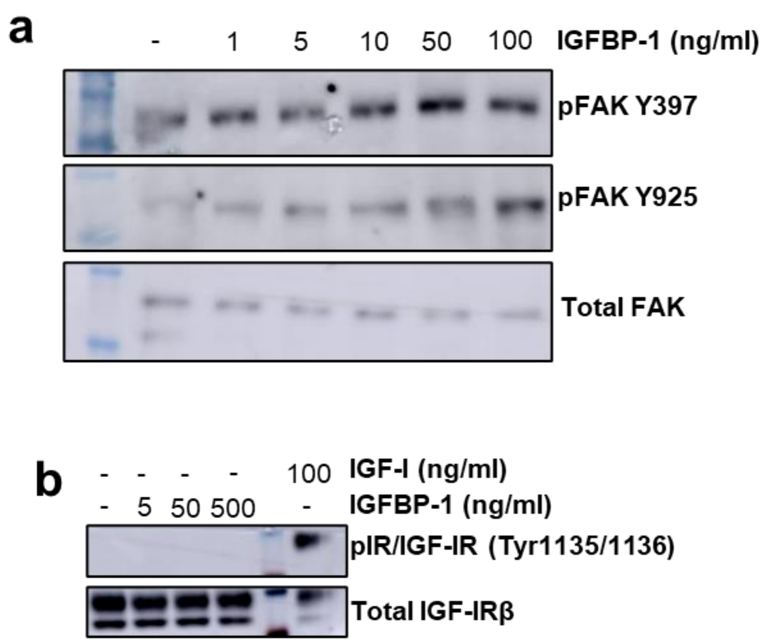

ESM Fig. 10: IGFBP-1 causes an IGF-IR-independent increase in FAK phosphorylation in human podocytes

Human podocytes were starved of serum (including IGF) for 2-4 hours before IGFBP-1 stimulation. (a) An increased phosphorylation of FAK (pFAK Y397, Y925) is observed in human podocytes following IGFBP-1 stimulation, at the stated doses, for 15 minutes; (b) No changes in IGF-IR phosphorylation (Tyr1135/1136) are observed following IGFBP-1 stimulation at the stated doses, for 15 minutes.

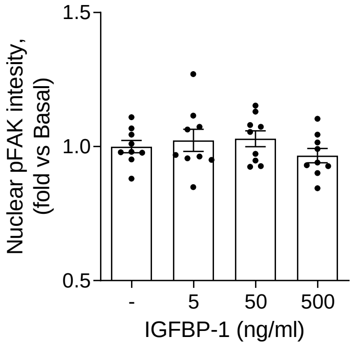

ESM Fig. 11: IGFBP-1 stimulation has no effect on nuclear FAK phosphorylation in human podocytes

Human podocytes were starved of serum (including IGF) for 2-4 hours before IGFBP-1 stimulation for 15 minutes at the stated doses. No significant change in the nuclear levels of pFAK (Y397) was observed at any concentration studied, One-Way ANOVA (p=0.47), n=3, 3 replicates per condition.

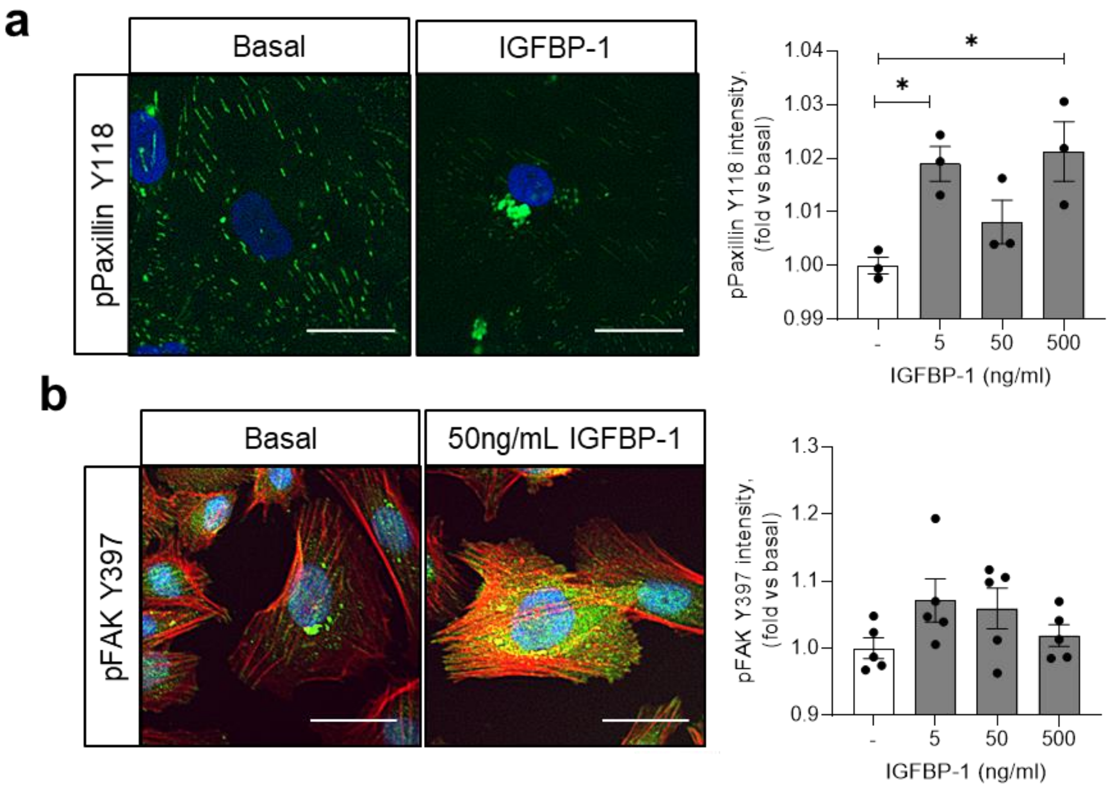

ESM Fig. 12: IGFBP-1 influences focal adhesion signalling in human podocytes

Representative fluorescent images (scale=50µm) and automated quantification of (a) pPaxillin (Y118) intensity in human podocytes treated with IGFBP-1 at the stated doses for 15 minutes, (n=3 experiments, 3 replicates per condition, *$p<0.05$, One-Way ANOVA with Tukey's multiple comparison test) and (b) Representative fluorescent images and automated quantification of pFAK Y397 intensity in human podocytes treated with IGFBP-1 at the stated doses for 15 minutes (n=5 experiments), One-way ANOVA $p=0.1872$, unadjusted p-value=0.08 5ng/ml BP-1 vs control.

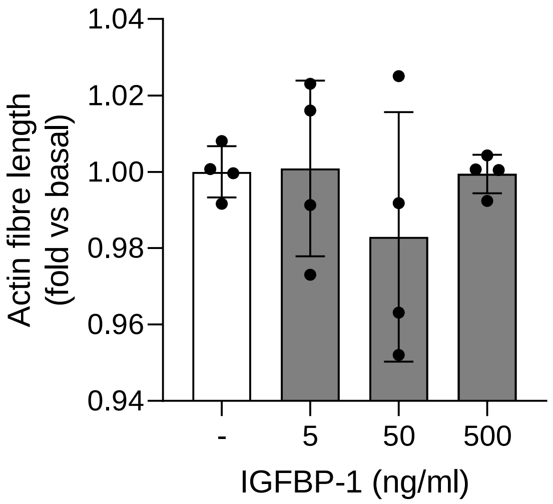

ESM Fig. 13. No consistent changes in the actin cytoskeleton are observed with 15 minutes of IGFBP-1 stimulation

Representative images and automated quantification of mean F-actin fibre length per cell in human podocytes stimulated with IGFBP-1 at the stated concentrations for 15 minutes (n=4, 3 replicates per condition, One-Way ANOVA (p=0.57) with Tukey's multiple comparison test, p=0.65 and unpaired t-test 50ng/ml vs basal p=0.35)

ESM References

[1] Weil EJ, Fufaa G, Jones LI, et al. (2013) Effect of losartan on prevention and progression of early diabetic nephropathy in American Indians with type 2 diabetes. Diabetes 62(9): 3224-3231. 10.2337/db12-1512

[2] Schmid H, Boucherot A, Yasuda Y, et al. (2006) Modular activation of nuclear factor-kappaB transcriptional programs in human diabetic nephropathy. Diabetes 55(11): 2993-3003. 10.2337/db06-0477

[3] Martini S, Nair V, Keller BJ, et al. (2014) Integrative biology identifies shared transcriptional networks in CKD. Journal of the American Society of Nephrology : JASN 25(11): 2559-2572. 10.1681/asn.2013080906

[4] Nair V, Komorowsky CV, Weil EJ, et al. (2018) A molecular morphometric approach to diabetic kidney disease can link structure to function and outcome. Kidney international 93(2): 439-449. 10.1016/j.kint.2017.08.013
